# Electric Power Grids Under High-Absenteeism Pandemics: History, Context, Response, and Opportunities

**DOI:** 10.1109/ACCESS.2020.3041247

**Published:** 2020-11-30

**Authors:** Benjamin Wormuth, Shiyuan Wang, Payman Dehghanian, Masoud Barati, Abouzar Estebsari, Tiago Pascoal Filomena, Mohammad Heidari Kapourchali, Miguel A. Lejeune

**Affiliations:** 1 Department of Electrical and Computer EngineeringThe George Washington University 8367 Washington DC 20052 USA; 2 Department of Electrical and Computer EngineeringUniversity of Pittsburgh 6614 Pittsburgh PA 15261 USA; 3 School of the Built Environment and ArchitectureLondon South Bank University 4914 London SE1 0AA U.K.; 4 Operations Research and FinanceFederal University of Rio Grande do Sul 28124 Porto Alegre Brazil; 5 Department of Electrical EngineeringUniversity of Alaska Anchorage 3291 Anchorage AK 99508 USA; 6 Department of Decision SciencesSchool of BusinessThe George Washington University 8367 Washington DC 20052 USA

**Keywords:** Absenteeism, COVID-19, electric power grid, lock-down, pandemic, resilience

## Abstract

Widespread outbreaks of infectious disease, i.e., the so-called pandemics that may travel quickly and silently beyond boundaries, can significantly upsurge the morbidity and mortality over large-scale geographical areas. They commonly result in enormous economic losses, political disruptions, social unrest, and quickly evolve to a national security concern. Societies have been shaped by pandemics and outbreaks for as long as we have had societies. While differing in nature and in realizations, they all place the normal life of modern societies on hold. Common interruptions include job loss, infrastructure failure, and political ramifications. The electric power systems, upon which our modern society relies, is driving a myriad of interdependent services, such as water systems, communication networks, transportation systems, health services, etc. With the sudden shifts in electric power generation and demand portfolios and the need to sustain quality electricity supply to end customers (particularly mission-critical services) during pandemics, safeguarding the nation’s electric power grid in the face of such rapidly evolving outbreaks is among the top priorities. This paper explores the various mechanisms through which the electric power grids around the globe are influenced by pandemics in general and COVID-19 in particular, shares the lessons learned and best practices taken in different sectors of the electric industry in responding to the dramatic shifts enforced by such threats, and provides visions for a pandemic-resilient electric grid of the future.

## I. Introduction

Electric power grids, as the most complex man-made cyber-physical system to date, have been traditionally designed to operate reliably under normal operating conditions and withstand potential outage-inducing events. However, power grids are constantly confronting a wide range of unpredictable and highly-uncertain threats that could impede preservation of sustainable, reliable, and high-quality electricity needed to electrify modern society. Future societal growth and innovations are now hindered by (i) geographically-vast area of coverage translated into an expanded exposure of the electricity grid to a wide range of threats, (ii) the rushing arrival of uncertain renewables and the intensified stochasticity in the electric grid generation portfolios, and (iii) the elevated number and severity of the extreme natural disasters (e.g., storms, hurricanes, earthquakes, wildfires, etc.) and man-made cyber-attacks. Safeguarding the nation’s electric power grid and ensuring a continuous, reliable, and affordable supply of energy in the face of such extremes, commonly known as high-impact low-probability (HILP) events, are among the top priorities for the electric power industry. While some of these extremes are difficult to predict, the electric industry generally recognizes the need to build in system survivability to keep the lights on at all times and to develop analytical tools for online monitoring, detection, verification, and mitigation of such threats and many other emergencies that adversely affect the power grid operation and control. Resilience of the electricity grid is now more critical than ever to people’s well-being and every aspect of our increasingly-electrified economy.

**FIGURE 1.**
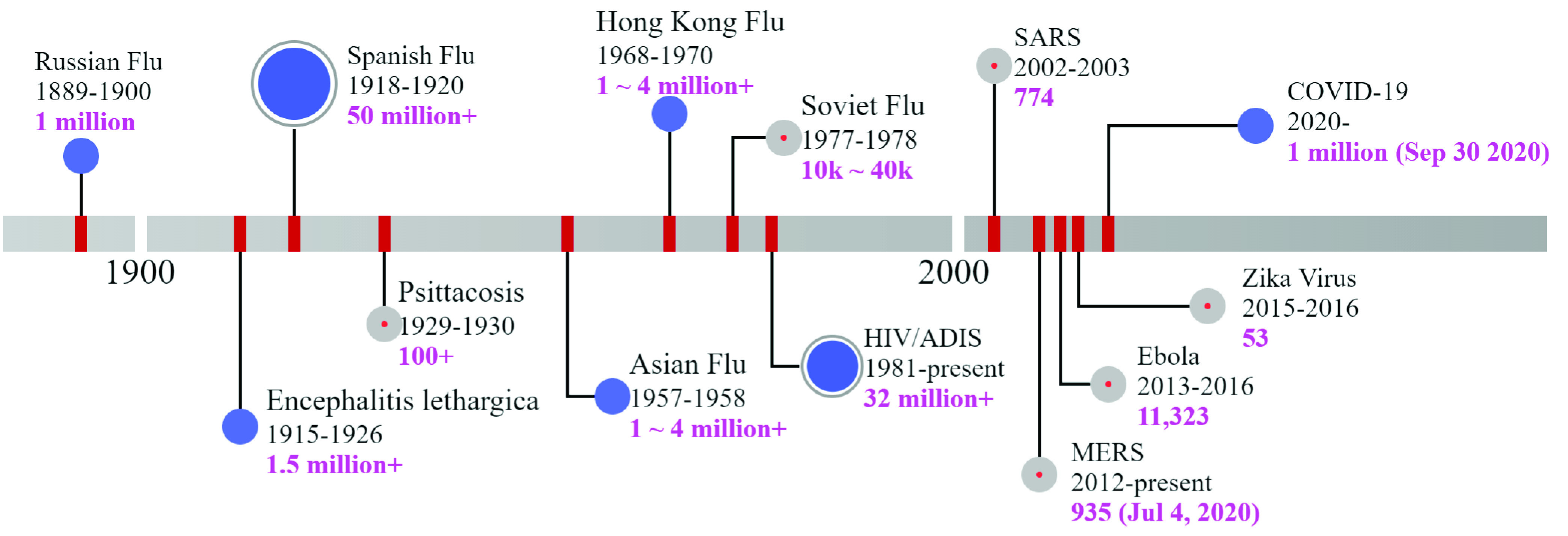
Global pandemics and deaths in the 20th and 21st centuries [1], [2].

The frequency of pandemics has been on the rise over the past 20 years (see Figure 1) [3]. Examples include the 2002 Severe Acute Respiratory Syndrome (SARS), 2009 Novel Influenza A Virus Subtype H1N1, 2003–2016 Ebola Virus Disease (EVD), and the ongoing Coronavirus Disease (COVID-19), with a large rate of fatality and occupation around the world. The emergence of pandemics is primarily driven by advancements in urbanization, intensified global travel and integration, drastic shifts in land use, and greater exploitation of the natural environment. While significant efforts and vigorous measures have been taken to identify and limit the emerging outbreaks that might lead to pandemics, various sectors of the modern societies and critical infrastructure around the globe are still vulnerable to and ill-prepared for these extremes. Pandemics have been observed to result in abrupt, co-evolved, and sometimes challenging shifts in socio-ecological ecosystems, businesses, and built environments [4].

Electric power grids are not an exception. The planning, operation and control of the grid is impacted by such “high absenteeism” events in different sectors and on multiple scales. From health emergencies that could rapidly and severely limit the available workforce in electric industry to supply-chain disruptions leading to postponed installations and delayed maintenance implementations, the electric industry has to take all necessary actions to (i) keep the lights on throughput the pandemic emergency and (ii) ensure the health and well-being of the available personnel and on-field workforce. Different from the business-as-usual practices, drastic changes and sudden shifts in the use of electricity by the industrial, commercial, and residential sectors during pandemics accrue the complexity of taking decisions under such highly volatile contexts.

This paper is a wide-ranging overview of the effects of pandemics on electric utilities, power grid, and load demand. By covering the effects of historical pandemics all the way through a plan in response to future pandemics, this paper provides context for the COVID-19 pandemic’s impacts on the utility industry and the demand for electricity. Drawing on all of these experiences, the paper aims to share the lessons learned and best practices in the fight between the power engineering and pandemics, shedding lights on what it takes to realize the pandemic-resilient power grids of the future.

The remainder of the paper is structured as follows. Section II provides background information on several major pandemics in the past 20 years and discusses how electric power grids responded to the resulting states of emergency. Focusing on the ongoing COVID-19 emergency, Section III presents a global perspective on how electric power grids of different sizes, technological complexity, and geographical classifications around the world have been influenced in 6 months of COVID-19 emergence and evolution. Section IV is devoted to the United States electric power grid and describes (i) the existing vulnerabilities in facing the outbreak, (ii) the actions and policies planned and/or taken by different authorities in the pre-pandemic alert phase and through-pandemic outbreak phase, and (iii) the post-pandemic plans in returning to the business-as-normal phase of operation. Section V introduces the observations on how the COVID-19 pandemic outbreak could impact the interdependent critical infrastructure and lifeline networks (e.g., communication networks, water systems, transportation networks, health sector, etc.) which are driven by the electric power grid and their response to COVID-19 pandemic could shift the electricity consumption patterns across the country. Section VI discusses the challenges and opportunities during and following the COVID-19 pandemic outbreak along with the visions for a pandemic-prepared and pandemic-resilient power grids. Section VIII provides the concluding remarks.

## II. Historical Pandemics and Electric Grids

COVID-19 is certainly not the first pandemic to pose challenges to the electric power grid. In the past 20 years, there have been a handful of outbreaks [3] that had the potential to disrupt society and damage the power grid operation (see Figure 1). The main stress on the utility system is absenteeism from workers getting sick [5]. Loss of institutional knowledge is a devastating risk during pandemics. Should enough workers die or become unavailable, the electric utility would have to spend massive sums of money to regain that knowledge. Until COVID-19, none of these past outbreaks had a widespread impact on the power grid operations and field practices. At each pandemic, electric utilities became more prepared to combat such events and revisit their contingency plans.

### A. SARS

In November 2002, the Severe Acute Respiratory Virus (SARS) was first discovered in the Guangdong Province in China. In February 2003, the virus resurfaced in Hanoi, Vietnam. In total, there were about 8,000 reported cases and 70 deaths worldwide [6]. With respect to the electric power grid, there was a large scare, but no major implications for reliability of the grid operation. The SARS epidemic has however a positive long-term effect in terms of improving pandemic preparedness. Many electric utilities either created or updated their pandemic preparedness plan in the wake of the crisis. By being proactive and creating a plan before the crisis, the utilities set the stage for a smooth transition through other future health crises.

### B. H1N1

After SARS, H1N1 emerged in 2009. It originated from Mexico and spread throughout the globe within a few weeks. Commonly referred to as the Swine Flu, the H1N1 outbreak caused between 151,700 to 575,400 worldwide deaths in the first year [7]. For comparison, as of 28 September 2020, the COVID-19 pandemic has caused 1 million reported deaths worldwide [8]. In 2009, the H1N1 outbreak was not severe enough to stress the power grid, so it did not provide an opportunity to test the infrastructure and pandemic plans for resilience. Apart from the deaths worldwide, the only result of this pandemic was some school closures and public concern for workers under 65 years old. Unlike other pandemics, 80% of the deaths involved people under 65 years old [7]. The majority of the workforce is under 65 years of age, so this meant special precautions needed to be taken for worker safety. Because of the SARS scare earlier, many electric utilities already had necessary pandemic plans in place. Utility work is often conducted in teams for safety reasons. This continued throughout the H1N1 pandemic for many utility companies. Efforts were made to sequester the field crews from the outside public and other utility workers (such as engineers and managers). One utility even had their field crews add a second barrier to increase the safety radius between the construction work and the public [9]. This helped isolate crews and prevent infection between the public and field crews. Utilities also feared high absenteeism and poor communication methods would hamper their work, but there are no documented instances of this being an issue during the H1N1 pandemic. High absenteeism is an issue because getting work accomplished is very difficult when key stakeholders are out sick at differing times. In severe crises, work may go unfinished if there are not enough healthy workers to complete the projects. Communication platforms have vastly improved since the 2009 H1N1 outbreak, and there are now numerous video chat platforms for communication if workers are working remotely. In many ways, the H1N1 pandemic improved the utility’s perspective and response to the COVID-19 pandemic. H1N1 served as a dry run for what was to come, and many electric utilities were able to simply update their H1N1 pandemic response plans for COVID-19.

### C. Ebola

From 2014-2016, 11,310 people died due to an outbreak of the Ebola Virus Disease (EVD) [10]. Primarily concentrated in West Africa, lack of access to reliable electric power made treating EVD particularly challenging. Electricity is crucial to maintain refrigeration, lab work, and ventilators. Without reliable power access, fighting a pandemic is exponentially more challenging. EVD is still a risk in Africa, and fighting it is still a challenge without reliable access to electricity. Many countries in Africa rely on rural micro-grids which lack the reliability found in larger grids.

### D. ZIKA

With the Zika outbreak during 2015 and 2016, there was not much impact recorded on utilities and the power grid operation. According to Eric Silagey, Florida Power and Light CEO, the only major change to business as usual practices involved encouraging outdoor workers to wear long sleeves, and increasing the use of bug spray. Because Zika was not usually fatal, it did not have as much impact on electric utility operations [11].

## III. COVID-19 and Electric Power Grids: a Global Perspective

Across the world, demand for electricity has mostly decreased amid the COVID-19 pandemic. With lock-downs in place and people working remotely from home, electricity usage was observed lower than usual. Even with many people staying home during the lock-down period, residential power usage only increased by 2.4%. It takes an extraordinary amount of energy to heat, cool, and light the large office buildings found in cities. With high-rise buildings in downtown areas, it takes more energy to pump water and run the elevators. While these energy usages may seem trivial, the aggregated demand for electricity has drastically decreased, and the results have been felt worldwide.

This section discusses the response of the electric industry to COVID-19 across the globe. While the United States has had the most deaths of any country, the virus has affected the electric power grid in other countries and continents in unique ways. This section analyzes such impacts in Canada, Asia, Africa, and the European Union.

### A. Canada

In March 2020, Ontario’s Independent Electricity System Operator (IESO) released an updated version of their pandemic plan outlining the response levels for different transmission rates of COVID-19 [14]. The document focuses on the resilient delivery of electricity at all levels, from generation to distribution. Canada had no major disruptions to its electric grid during the COVID-19 outbreak, partly because of the extensive planning. Planning documents have helped utilities keep information well documented and inform employees about all it takes for the lights to be kept on during the COVID-19 emergency [14]. Electric utilities are skilled at planning for emergencies and crises. Many have extensive plans in place for natural disasters, and many have employees holding both a blue-sky job and a storm-duty job. This allows them to mobilize their workforce to restore power as fast as possible. This also instills a culture of flexibility into the employee’s work. Employees understand that their sole goal above all else is to keep the lights on. This is a very important culture to maintain during a pandemic as employees work from home and possibly become disconnected from their company. Ontario IESO’s document is proof that the Canadian utilities were prepared for the pandemic.

In terms of the load demand, Canada experienced a drastic reduction in power consumption during the strictest lock-down phase of the pandemic. In Ontario, there was a drop of 1000–2000 MW of load demand, or about 10% of their regular demand [17]. This is equivalent to the city of Ottawa no longer using power and imposed serious challenges on the grid. Chiefly, power generation has to be significantly reduced. While this is possible, it places new challenges on the ISO operator during an already stressful period. Generation sources have to be significantly throttled. The grid is highly dynamic within its limits, but such drastic drops significantly stresses the system.

**FIGURE 2.**
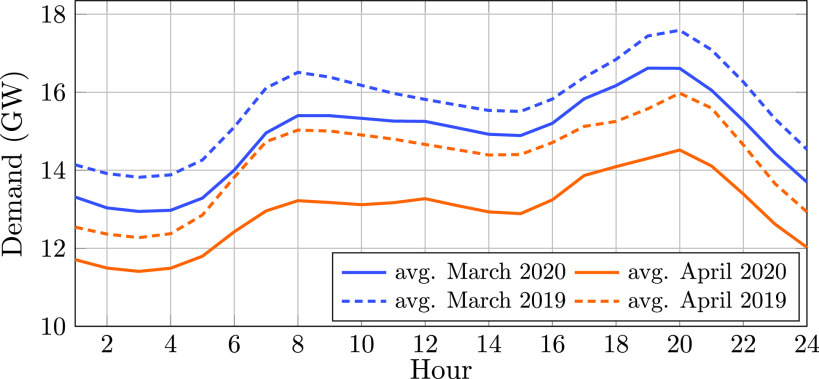
The load softened and shifted slightly later in the day due the COVID-19 pandemic [13].

In addition to lower load values, the traditional duck curve for daily power consumption in Ontario, Canada has shifted (see Figure 2). While the traditional curve begins to rise around 7 AM, the curve now rises later in the morning [13], [17]. In addition, the evening peak has flattened as compared to the traditional setting where there would be a peak around 5–6 PM as people get home from work. These changes can challenge the system operators responsible for ensuring a reliable delivery of power to end-use consumers.

### B. Asia

**FIGURE 3.**
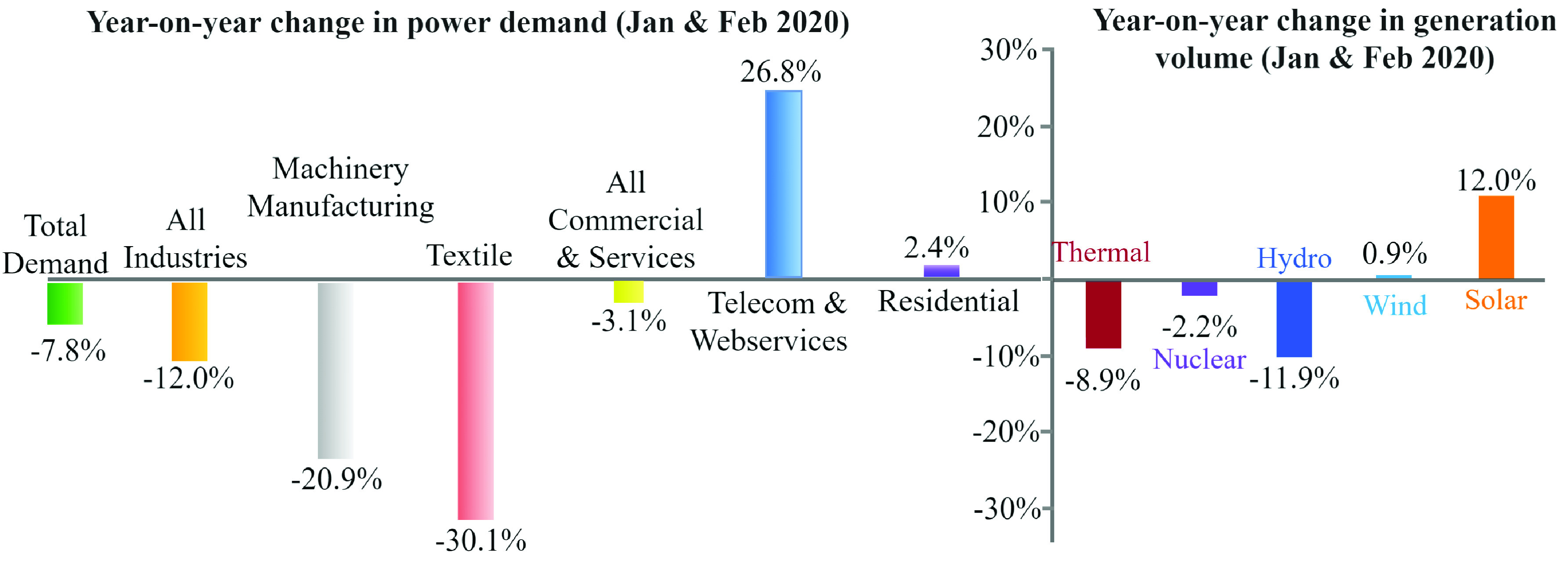
Electricity markets in China during a COVID-19 lock-down period [12].

The first confirmed cases of COVID-19 appeared in Wuhan, China in mid-January 2020. Almost immediately, the affected areas locked down. According to the IHS Markit [12], the electricity demand dropped 7.8% for January and February throughout China. Electricity use in industrial facilities and textile industry dropped around 12% and 30%, respectively (Figure 3) [12]. The response was felt primarily in the Hubei province, and the electricity demand remained fairly resilient outside of the originating province. By April, the demand for electricity again ticked upward nationwide [18]. Such large swings in industrial power usage impact the power grid and the utility operations because electric utilities usually charge large industrial customers for both the real and reactive power delivered. Residential customers are only charged for their real power usage, so even if the demand was an equal shift from industrial to residential customers, some electric utilities would still be earning less than before.

**FIGURE 4.**
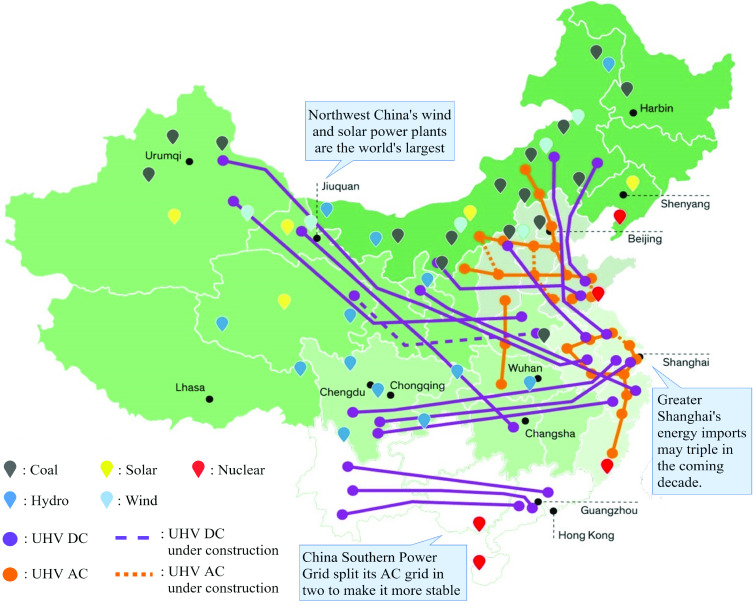
Road-map for HVDC transmission lines in China’s national power grid [15].

Following the first pandemic surge, China is poised for a massive economic growth in the renewable sector [19]. COVID-19 has pushed governments further towards unprecedented debt levels, and this will no doubt impact the transition to renewable energy sources. The International Energy Agency (IEA) estimates that the global solar photovoltaic deployment will decline around 8% in 2020 due to the financial crunch the pandemic has caused [18]. At the end of the day, renewable deployment is still expensive for governments, and the structural upgrade of the electric grid is seen as non-essential during a global pandemic. In China, however, the transition to renewable energy sources has continued throughout the pandemic. China manufactures many of the world’s solar panels. In addition, solar generation grew 12% year over year [12]. Chinese companies will experience massive growth in the following years in part because countries like India have delayed solar installation. In the coming years, these delays will drive up demand for renewable solutions. China has focused most of its recovery efforts on maximizing Gross Domestic Product. In March 2020, State Grid Corporation of China, China’s national power grid, announced its investment of $26 billion into installing High Voltage Direct Current (HVDC) lines throughout the country [21] (see Figure 4). Some of the current HVDC projects in China are shown in Table 1. HVDC lines allow for more remote renewable power generation such as wind and solar, since increased HVDC capacity allows for power transfer over longer distances with lower power losses.

**TABLE 1:** Current HVDC Projects in China [16]

Project	Capacity	Length (km)	Starting Location	Ending Location	Completion Date (estimated)
Shaanxi-Hubei Line	14 GW	3,000	Shaanxi	Hubei	2021
Yazhong-Nanchang Line	8 GW	1,700	Yazhong	Nanchang	unknown
Shaanbei-Wuhan Line	8 GW	1,100	Shaanbei	Wuhan	unknown
Qinghai-Henan Project	8 GW	1,500	Qinghai	Henan	unknown

**FIGURE 5.**
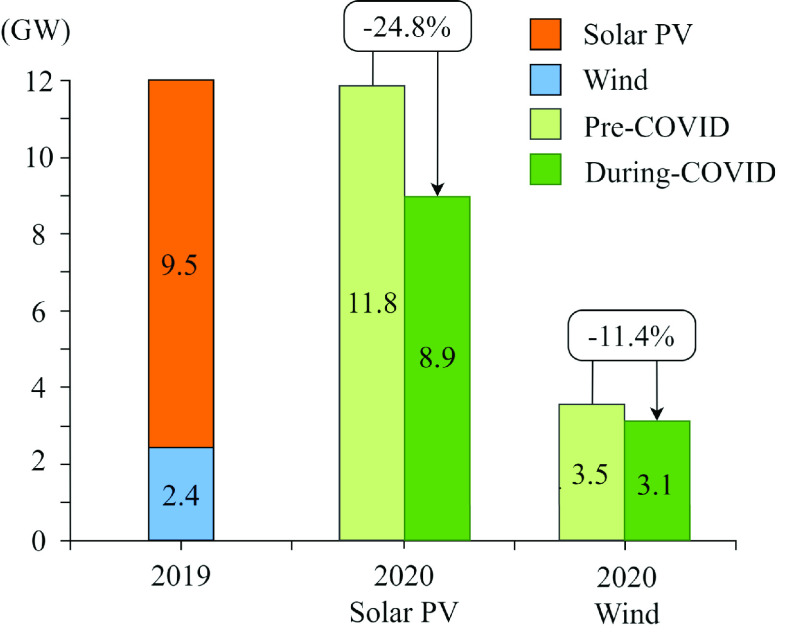
Expected New annually-added installations: pre-COVID (2019) vs. during-COVID (until second half of 2020) in India [16].

In India, the ongoing pandemic has delayed an estimated 3000 MW of solar and wind deployment across the country which were planned to be achieved in 2020 (see Figure 5) [20], [22]. Delaying renewable energy installations is a phenomenon that is seen in numerous countries over the past 6 months of COVID-19 progression. Many renewable projects are partially government-funded, and the government funding for energy projects dried up with the global pandemic. The capital investments for renewable energy implementation was moved to funding hospitals and health care services, taking care of the sick, and supplying personal protective equipment, such as masks and gloves.

### C. Africa

Sub-Saharan Africa is facing unique challenges with its power grid. According to the World Bank, “*only 28% of healthcare facilities have reliable electric power, and only 43% of the population is electrified at all*,” posing unique challenges for health care officials [23]. Like under Ebola, necessary health measures during COVID-19 are challenging to implement without reliable electric access. That being said, much of Africa has fared comparably well under the COVID-19 pandemic. Many African countries have lower case numbers than the rest of the world, except for some localized hot-spots in Gabon, South Africa, and Libya [24]. Research is ongoing to determine reasons and the impacts.

**FIGURE 6.**
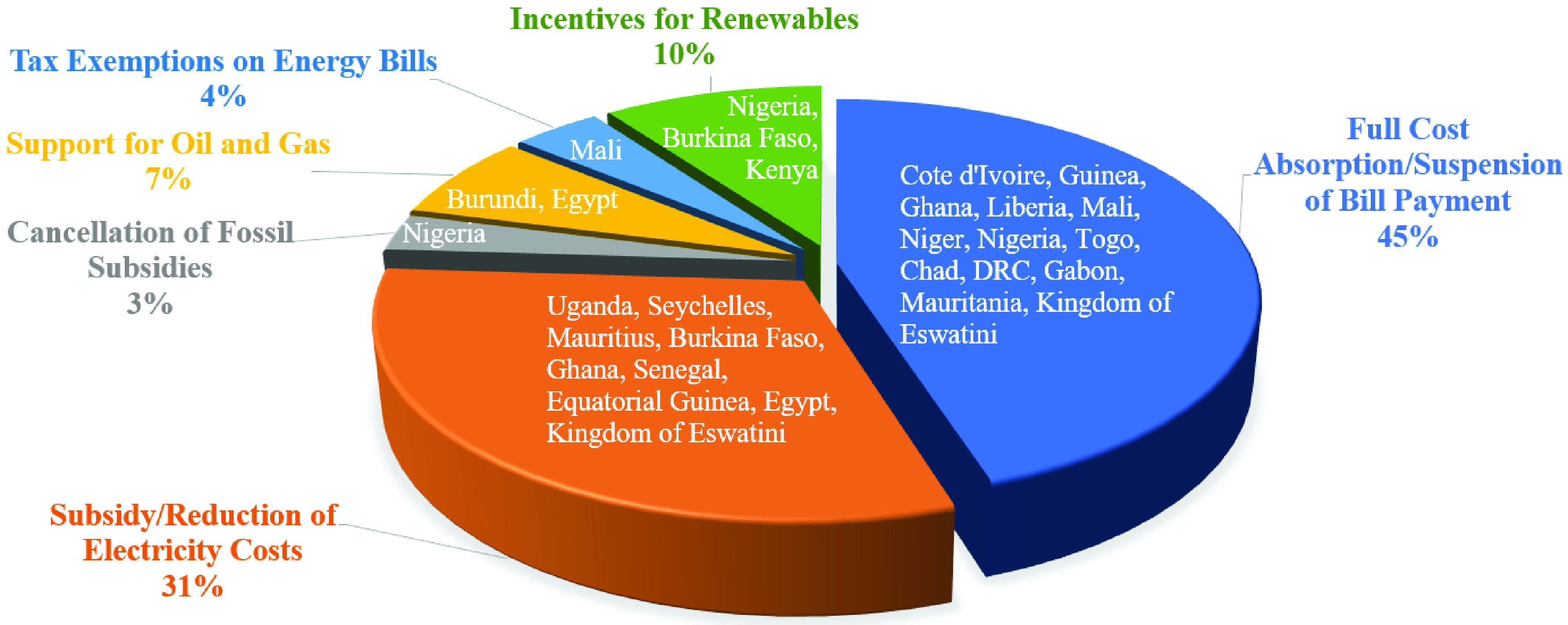
Summary of support measures provided across Africa for energy sector reinforcement during COVID-19 [20].

Another challenge for Africa is the implementation of off-grid solar projects. Some of these projects require outside investment from capital investment firms beyond its continental boundaries. With the COVID-19 being a global pandemic with severe economic implications, there is a chance that those firms will less likely be able to fund projects. Thus far, this has not been the case, but the pandemic may pose long term challenges if investment capital becomes harder to find. In many African countries, the government has stepped in to help citizens manage their electricity bills, and to help utility companies stay in business during COVID-19 pandemic. Support measures range from support to oil and natural gas to subsidizing residents’ energy bills. Figure 6 shows the various support measures throughout the continent. In total, 23 support measures have been implemented.

### D. Europe

Europe was considered the center of the global COVID-19 outbreak by the World Health Organization (WHO) as of March 13, 2020. In response to the pandemic, different European countries took different approaches. Some countries like Spain, Italy, Belgium and the UK applied severe restrictions, while a few others like the Netherlands and Sweden took less restrictive measures. In the following sub-sections, the impacts of different containment measures on demand for electricity, renewable installations, and electric industry personnel, are discussed.

#### 1) Impacts on Demand for Electricity

**FIGURE 7.**
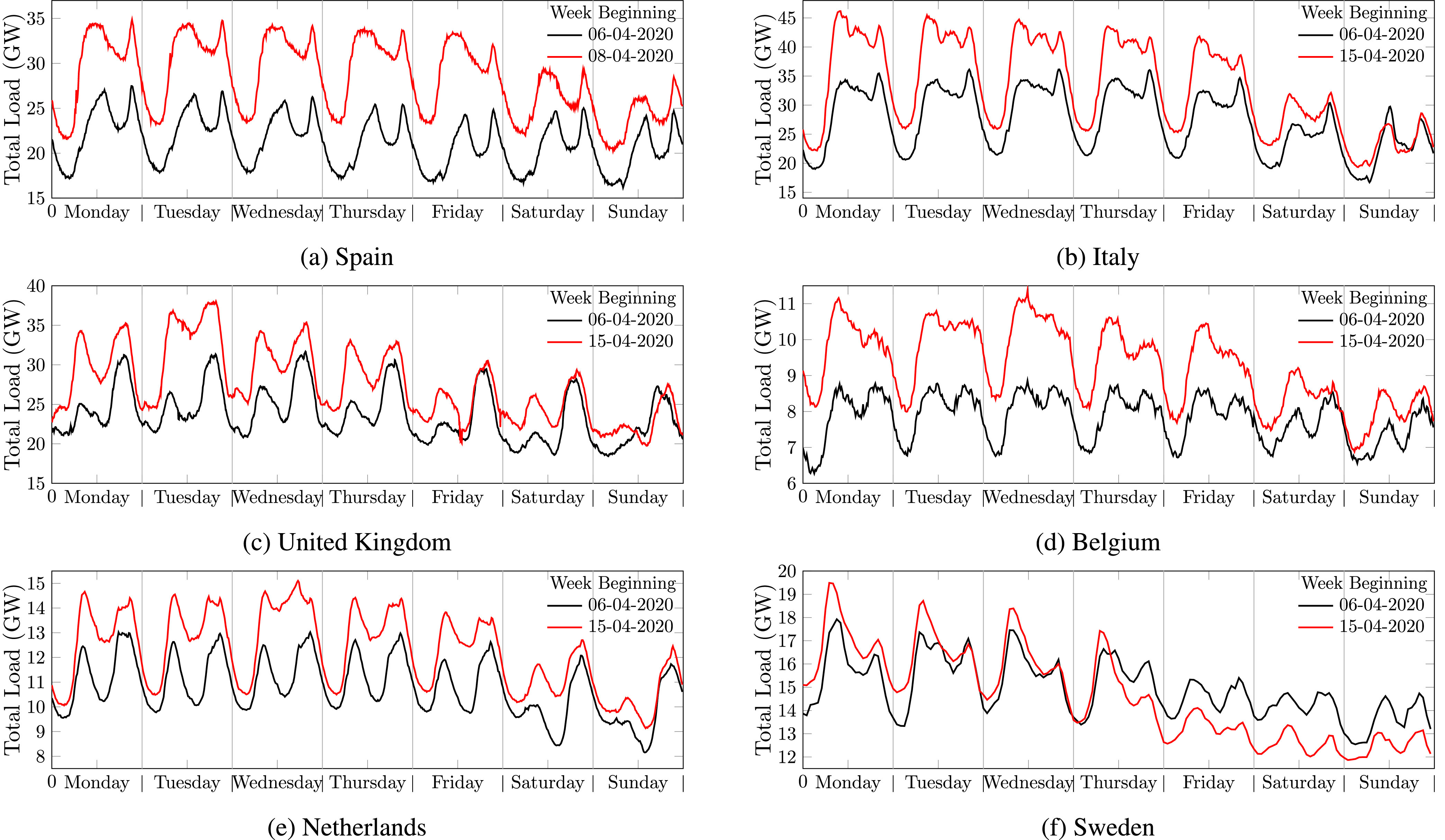
Electricity consumption during the second week of April 2020 compared to a reference week in 2019 for (a) Spain [25], (b) Italy [26], (c) the UK [27], (d) Belgium [28], (e) Netherlands [29], and (f) Sweden [29].

Comparing the electricity consumption profiles in different European countries reflects the impact of various measures on the households’ activities and electricity demand [30]. Figure 7 compares the weekly electricity load consumption in some of the European countries amid COVID-19 measures with a reference week in the year before with a comparable daily average temperature. Except in Sweden with the lowest restrictions in Europe, electricity demand substantially decreased in all other countries after the pandemic was declared and restrictions were implemented. Although lock-downs made the residential load higher than during the normal working days, closure of small businesses with commercial and industrial loads remarkably decreased the total electricity demand. In Belgium, the transmission system operator reported that the loads connected to the power distribution system were reduced more noticeably than those directly connected to the transmission system [31].

Authors in [30] used a Demand Variation Index (DVI) as shown in (1), to compare the average reduction in electricity demand to a reference period.

\begin{equation*} DVI = \frac {\sum ^{n}_{i=1}(P^{old}_{t_{i}}-P^{new}_{t_{i}})}{n\times \overline {P^{old}}}\times 100 \tag{1}\end{equation*}

where $P^{new}_{t_{i}}$ is the load at time $t_{i}$, $P^{old}_{t_{i}}$ is the load at the same time in the reference year, $n$ is the number of load samples, and $\overline {P^{old}}$ is the average demand during the reference period. The DVI for the countries under this study including Spain, Italy, Belgium, UK, the Netherlands, and Sweden, were calculated as 25%, 17.7%, 15.6%, 14.2%, 11.6% and −2.1%, respectively. This shows that Netherlands with less restrictions had a low variation compare to the reference period and Sweden with the least restrictions had even a slightly higher electricity demand.

The changes in load patterns are more marked for the daily domestic demand. The reduction in the commercial and industrial demand affects the short-term and mid-term load forecasts, on which electric utilities rely for planning. For example, based on the forecasts, utilities perform reconfiguration in power distribution networks to decrease the network losses, to improve the voltage profile, and to prevent overloading conditions. With such changes in load profiles, the reconfiguration plans need to be reconsidered. Increased losses and inappropriate voltage profiles may be unavoidable in some cases. As the demand for electricity reduces, the share of renewable energy resources increases. However, electric utilities may need to curtail them to avoid overvoltage conditions across the power network.

#### 2) Impacts on Renewable Installations

In Italy, the electricity demand dropped between February 21 and March 21 by more than 18% as the lock-down was imposed on the country [32]. Italy experienced a slower economic recovery than most other countries in the European Union, partly because of the stringent lock-downs making it difficult to return to business as usual. Italy was also one of the main hot spots in Europe, meaning that they had a lot of COVID-19 cases and the virus was centered in the country for a while. In May 2020, the Italian government raised the tax deductible benefit for solar panel installations from 50% to 110% in a bid to encourage solar installations and get the installers back to work. Like most other countries, Italy experienced disruptions in such renewable energy plans [33].

In July 2020, the EU experienced 5% lower demand for electricity than a year prior. According to the IEA, across the EU “lock-down periods have seen new highs in variable renewables contribution as a share of electricity demand [34].” This increase in renewable energy usage is due to multiple reasons, but partly because the overall demand decreased. Lower demand allowed for a higher percentage of renewables to be used in the power generation portfolio.

#### 3) Impacts on Electric Industry Personnel

The major impacts of COVID-19 in Europe are felt on the workforce and absenteeism. Power system companies are aware of such effects and established lock-down or teleworking measures for their personnel. In France, just several days after the pandemic outbreak, three Électricité de France S.A employees in nuclear power plants tested positive for coronavirus. Several others who were in contact with those employees were asked to self-isolate for at least two weeks. In Croatia, the main power company advised all employees to perform all activities they can at home. In Belgium, 95% of employees of the transmission system operator Elia started working from home. Some essential employees who had to work from their offices must comply with the enhanced hygiene measures. Power network operators were also halting travels and limiting in-person meetings. The employees of 50Hertz, a German transmission system operator, were no longer allowed to travel to areas classified as risky, and their private travels were also banned. This would substantially impact enhancement and maintenance plans. The control room and other essential personnel were able to operate self-sufficiently [35]. In Greece, the main electricity company named PPC advised its employees not to commute to work by public transportation. They only allowed maximum of 5 customers to enter public offices at the same time. In Slovenia, ELES, the transmission system operator isolated a team of six employees at the national control center for two weeks. They would then be replaced by colleagues who were in home quarantine. Most power companies were following governments’ new regulations to protect their employees against COVID-19, and this has resulted in a large percentage of a company’s employees working from home. The limitations in work-space, communication challenges, lack of IT skills in senior staff, and possibility of cyber-attacks, is affecting the performance of electric utilities and power companies across the Europe and possibly worldwide.

## IV. COVID-19 and the U.S. National Power Grid

### A. Impacts on Electric Industry Personnel

When the COVID-19 pandemic first appeared in the United States, electric utilities were impacted like every other industry. Most non-essential workers were sent home and essential workers masked up to protect themselves at work. This is the same trend seen across almost all other industries in the U.S. The non-essential workers sent home, however, are essential in the long term. Even though their services are not required for the day-to-day operation of the electric grid, their absence will be noticed in the long run. The engineers and managers sent home are responsible for maintenance, testing, and design of modern power grid facilities such as substations, protection schemes, and service. Many of these employees hold certifications in their roles, and such personnel cannot easily be replaced [5]. Recruiting for these positions is also challenging. Upper level engineers are almost exclusively pulled from other electric utilities, but this becomes challenging if every utility is experiencing the same pandemic and shortages. Furthermore, recruiting younger entry-level engineers becomes a challenge. Many of these positions are filled through internship programs or college recruitment fairs in the U.S., and these events were mostly cancelled during the COVID-19 pandemic. Additionally, the loss of critical employees poses challenges in storm response, maintenance, and service planning.

### B. Impacts on Power Generation Sector

Pandemic planning usually revolves around securing the transmission system against the threats and absenteeism. Little attention is given to securing the fuel supply, even though this is a critical component to power generation [36]. We here discuss aspects of the power generation sector that would be impacted the most during a high-absenteeism pandemic: (i) fossil fuel supply chain and (ii) renewable energy investment, deployment, and operation.

#### 1) Pandemics and the Fossil Fuel Supply Chain in the U.S

The fossil fuel chain in the United States is surprisingly fragile, and there is not much coal held in reserve should the supply dry up. Even though there is a large push towards renewable energy resources, many electricity markets near coal mines still burn coal as it is the cheapest fuel available [36]. 40% of the nation’s coal production in 2018 came from the low-sulfur mines in Wyoming’s Powder River Basin (PRB), which yielded 304.2 million short tons. The total coal production in the Western region amounts to 418.3 short tons, or about 40% of the total US coal production (756.2 short tons) [36], [37]. Most of this coal is hauled by train to distant power plants, some as far away as Georgia. A high-absenteeism interruption in the transportation sector may disrupt the supply chain in a coal-driven electric industry. This may have severe implications in the electric utility finance as, under such disruptions and fuel availability uncertainties, some entities might have to take actions such as buying electric power from other utilities, reducing generating time, and buying coal from other sources. During the 1918 pandemic, the coal supply was disrupted due to illness. Granted, the country relied far more on coal plants for electricity then, but this still indicates it is a serious challenge for coal-fired electricity markets. In New York City, it has been reported that anthracite shipments dropped about 17% from their typical levels [36].

Current pandemic plans will not be enough to sustain the coal supply chain. Although coal is being used less and less for electricity generation, it is still a crucial component in the power generation portfolio for many energy markets. Coal workers are essential personnel working in close quarters, and they are usually considered last for preventative health measures. The coal supply chain should be given a higher priority in future pandemic planning to ensure reliable power supply. In addition to coal, nuclear is another source of power generation where there are significant deficiencies. Should a number of nuclear workers become ill or unavailable due to a pandemic, running nuclear reactors successfully may be a challenge. Hundreds of steps are necessary for nuclear refueling, and the workers who perform these tasks are highly specialized, the absence of whom may significantly endanger the electric industry [36]. Many organizations in the electric sector are highly dependent on contracted employees and vendors for essential support functions. Breach of contract situations may occur during a pandemic, which would further impact the affected entities. Cross-sector interdependencies will also affect the electric sector in the event of a pandemic.

At the national level, variations in electricity demand may have severe economic effects. If power generation exceeds the demand for electricity, electric utilities need to pay customers to increase further the electricity consumption and to avoid system imbalance and failures. This means that electricity producers may have to pay consumers to purchase their electricity. Negative electricity prices are a result of the lack of flexibility of the conventional generation system [39]. However, the price would be also affected by fuel price fluctuations due to the considerable reductions experienced during the COVID-19 pandemic and major producers’ disagreements.

#### 2) Pandemics and Renewable Energy in the U.S

**FIGURE 8.**
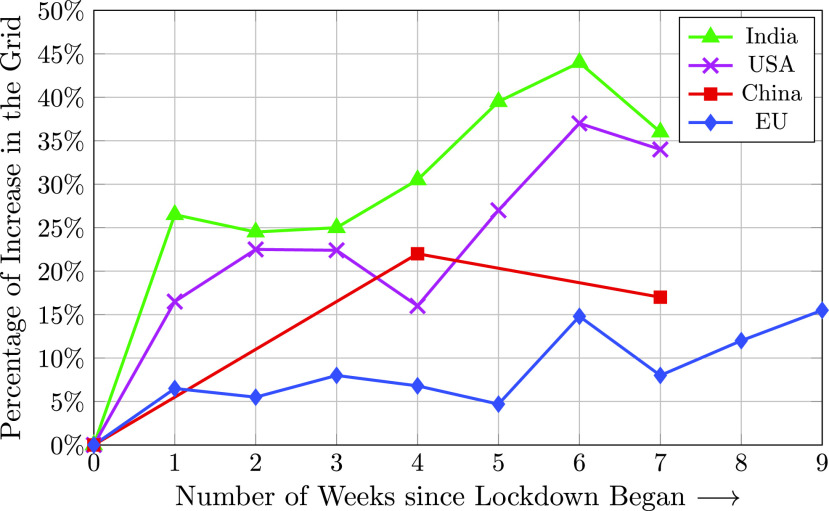
Renewable energy demand following COVID-19 lock-downs [38].

Demand for renewable energy resources under the pandemic has been increasing. While solar and wind installations have dropped around the world, the percentage of wind and solar utilization in the power generation portfolio is at an all time high. Renewable energy production and utilization is not dependent on the current pandemic, but only dependent on the weather variations. This means that generation sites are able to throttle down their nuclear and coal generation when the sun is shining. In fact, according to the U.S. Government’s Energy Information Administration (EIA), renewable consumption in the U.S. has passed coal for the first time in 130 years. During the COVID-19 lock-down, the electricity demand dropped greatly allowing renewable percentages to increase. In the US, renewable demand increased by 40% [38], [40] (see Figure 8).

More renewable energy in the generation mix reflects higher volatility, and it is harder to forecast power generation into the future. Most power in response to demand for electricity is purchased the day before or more, so having the wind be lighter than expected or having more clouds in the sky forces electric utilities to purchase power immediately. This power is far more expensive than power purchased in advance, thereby hurting the electric utilities financially. An alternative strategy is throttling renewable energy and keeping nuclear and coal constant, but at the expense of environmental challenges and higher operating costs.

The IEA forecasts that the net renewable energy additions would decline by 13% in 2020 compared to 2019 [41]. According to the Wood Mackenzie, an energy analysis firm, solar and wind installations are expected to decline 20% and 6%, respectively, this year [42]). It is expected that these numbers will rise again as the pandemic subsides and governments try to make up for the lost time. A massive influx of capital into renewable energy is expected in the coming years. Low operating cost and incentives from all sides for green energy is likely to keep the interest in renewable roll out and implementation very high [38].

### C. Impacts on Power Transmission and Distribution Sectors

Pandemics harbor unique threats to the electric grid. Illness and disease alone will not cause power lines and transformers to malfunction and result in a fault scenario, but they can negatively affect the system operators and the way how the grid is operated. The primary risk involving pandemics and power grids is absenteeism for critical system operations. To deal with this issue, many electric utilities publish their own guides for pandemic response, like Ontario’s planning document [14]. These documents are periodically updated with the latest science and thinking, so utilities simply have to dust off the latest version when the plan is put in place. In these documents, plans, procedures, and essential personnel are laid out. These plans also lay out plans for how work will continue with proper safety measures.

At National Grid, a utility servicing the Northeastern United States, maintenance work is continuing as normal with proper social distancing and masks. The power line crews are still continuing their work, albeit with social distancing. Engineers work from home, but they still complete field visits when necessary. Utility contractors doing more of the routine tasks have had their travel reduced, and they are doing more jobs closer to their home. This reduces the geographic spread of the virus. Utility maintenance workers, by virtue of working outdoors, is a low risk profession for virus transmission. Like many utilities, National Grid implemented strict requirements for their System operators inside the control room. Starting in March 2020, 200 workers were sequestered in a secret location. Most employees lived in the company provided recreational vehicles with access to catered meals and basketball courts. At the end of the month, a second group of 200 workers was cycled in. In between groups, the facilities were thoroughly cleaned. This extreme living arrangement helped reduce the spread of COVID-19 [43]. In addition to utility preparations, NYISO, the independent system operator (ISO) in New York, was sequestering employees on site in response to COVID-19 threat and to reduce the spread of the virus. By keeping people in a closed bubble, there is less worry about the need for testing and social distancing. All NYISO employees would still be social distancing, however, to be as safe as possible [44]. Other ISOs had similar plans in place and have implemented such practices during the past 6 months of the COVID-19 evolution.

In addition to the utility and ISO preparations, the North American Reliability Corporation (NERC) has made preparations for reliable power transmission. NERC relaxed some of their requirements and audits for electric utilities while there is a pandemic going on. In coordination, the Federal Energy Reliability Council (FERC) announced similar relaxations. FERC also announced a plan to expedite COVID-19 related requests, and they eased some requirements for in-person meetings [45].

The other challenge for the U.S electric utilities is managing Capital Expense (CapEx) and Operation Expense (OpEx) budgets. CapEx budgets allocate money for capital improvement projects like new substations, pole replacements, and new transformers. OpEx budgets are allocated for rent, payroll, and research costs, among others. Over 2020, CapEx budgets are decreasing because there is less equipment to install and the pace of work has slowed. While this may seem positive, in the complicated world of utility budgets, this is a challenge. Electric utilities should spend as close to their respective CapEx and OpEx budgets as possible. If the budgets get out of alignment, rate filings get harder and the utility is less likely to get their requests approved. This also increases OpEx budgets in subsequent years due to aged equipment in need of repairs.

Besides the above challenges, continued overloads in residential feeders and transformers may lead to equipment aging and subsequent increased failures. Electric utilities usually have maintenance planning for routine inspection and servicing to keep components in a reasonably functional condition, to maximize their life-cycles and to avoid future failures. However, with considerable variations in load profiles, the scheduled maintenance is not optimal, and rescheduling may be required. Moreover, due to limitations in the number of field crew during a pandemic, maintenance based on regular schedules is difficult to carry out.

### D. Pandemics and Demand for Electricity

Power demand in the US has taken a hit by the ongoing COVID-19 pandemic. As in the rest of the world, electricity usage has shifted from industrial to residential customers. Unlike Europe, comparing load demands on an aggregate scale in the United States is notoriously difficult. U.S. electric utilities do not report their load data to a central location. On a small scale, many utilities have reported drops in their load demand during the COVID-19 period. According to the Wall Street Journal, Snohomish County Public Utility District reported missing their energy targets when the COVID-19 lock-downs began. The utility serves numerous Boeing Factories and just over 800,000 people. Such uncertainties have challenged the energy managers and energy traders who need to forecast the future demand [32].

#### 1) Tools for Measuring Impacts of COVID-19 on Electricity Demand: Load Forecasting and Load Backcasting

**FIGURE 9.**
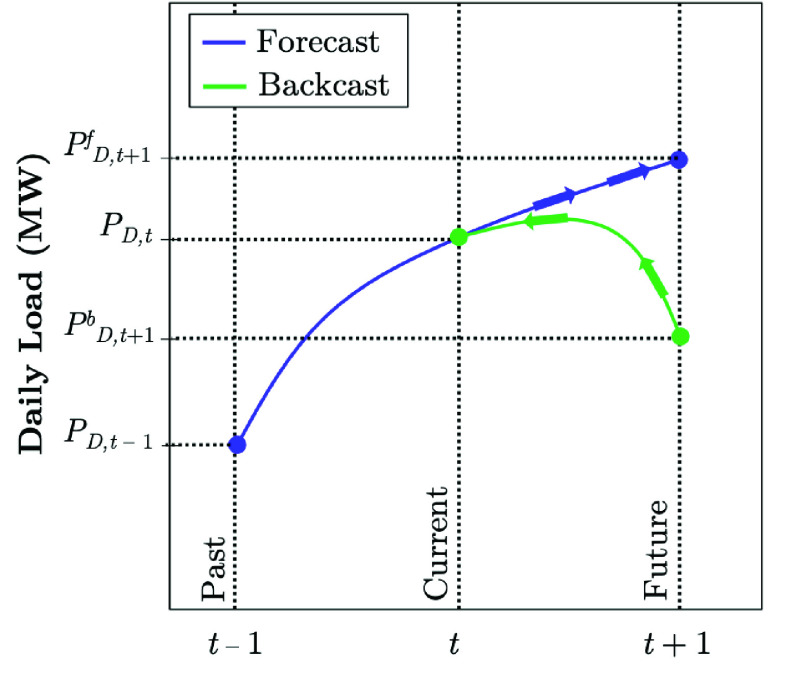
The implemented load forecasting and backcasting concepts for the ISO’s electrical demand prediction during COVID-19 lock-downs.

Multiple scenarios are commonly approached in the electric industry to investigate possible pathways on how to reach a desirable estimate of the electrical load consumption in the future. The *load forecasting* is the method of exploring future load that has not been yet observed or determined. The *load backcasting* typically refers to exploring the load realizations in the past, given the information known to date [47]. If the day-ahead temperature forecasts are used to provide the day-ahead electrical load forecasts, the results are ex-ante forecasts in the day-ahead load forecasting. If the actual temperatures of the next day are used, the results are ex-post forecasts. Therefore, ex-post forecasting is commonly considered as a subset of the backcasting [48]. Figure 9 visually describes the differences between the load forecasting and load backcasting methods. In the forecasting method, the present $P_{D,t}$ is the starting point for a future pathway until $P^{f}_{D,t+1}$. On the other hand, in the backcasting approach, $P^{b}_{D,t+1}$ describes a desirable future state. This may be a future load where there are very much lower electrical load consumption than today and/or lower electrical energy consumption levels in general [48]. The backcasting model is compelling for electric utilities and the ISO planning department in their disruptive discoveries and resource allocation practices. Using this tool, electric utilities and ISOs can envision a desired future, then plan backward to the present, making reasonable assumptions about emerging capacities, resources, and demand-side management practices.

#### 2) Early- and Recent-Stage Impacts of COVID-19 on Demand for Electricity: the Case of PJM

**FIGURE 10.**
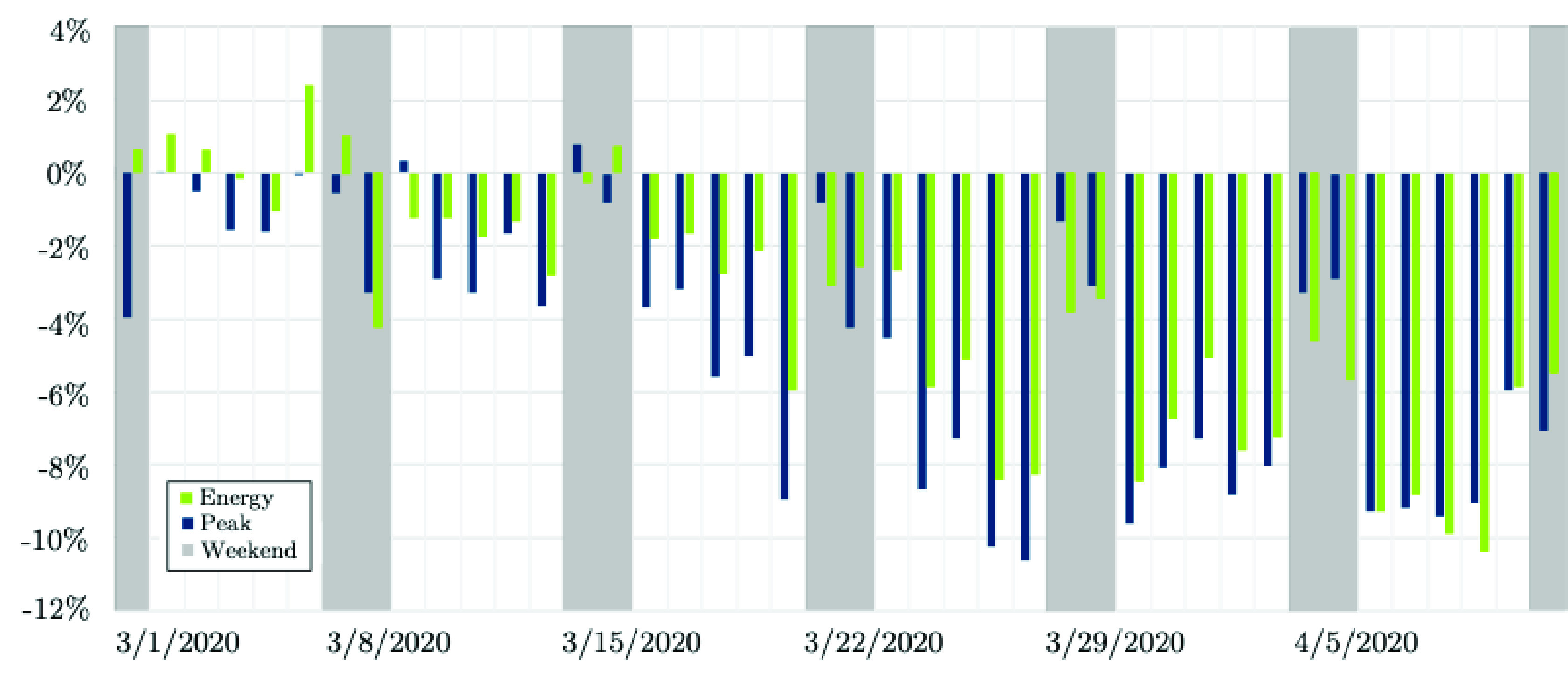
Early-stage impact of COVID-19 on the electricity demand: The case of PJM [46].

Figure 10 shows the “estimated” impact of COVID-19 on the peak and energy of the daily load consumption at the PJM ISO [46]. The estimation algorithm is the solution of the long-term load forecast model for each day using actual weather conditions [46]. As the actual behind-the-meter solar production data is not available in the PJM dataset, the model assumes the average behind-the-meter solar production based on the time of the year. This provides an estimate of what the load would have been for each day if COVID-19 did not exist ($P^{f}_{Dt}$). In order to compute the load consumption changes (in %), the MW difference between the actual load on each day ($P^{a}_{Dt}$) and the estimated load under actual weather conditions ($P^{f}_{Dt}$) is captured [46]. The estimated impact (in %) of COVID-19 on the electricity demand is given in (2).

\begin{equation*} \Delta P_{Dt} = \frac {P^{a}_{Dt}-P^{f}_{Dt}}{P^{f}_{Dt}}\times 100 \% \tag{2}\end{equation*}

As an example, the estimated load for April 2nd, 2020 from the forecast model is $P^{f}_{Dt} = 90,873$ MW. The average behind-the-meter solar adjustment for April 2nd is 1,049 MW, and the actual load realized on the same day is $P^{a}_{Dt} = 82,867$ MW. Therefore, the estimated MW impact of COVID-19 on electricity consumption measures is −8, 006 MW and the estimated percentage impact is found $\Delta P_{Dt} = \frac {-8,006}{90,873} = -8.8\%$. From Figure 10, it can be seen that during the second week of April 2020, the peak load came in on average 8–9% lower (~ 7,500 MW) than what the PJM anticipated. The largest impacts on the electricity consumption in the PJM service area were observed around 10-11% on March 26th and 27th (~ 9,500MW). On weekends, however, there are no significant changes other than slight reductions on the load consumption patterns. Weekends seem to have been impacted less (~ 2–4%). The load energy consumption was less affected, with the average weekday reduction since mid-March being 7% (~ 140 GWH per day) compared to 8% on peak.

**FIGURE 11.**
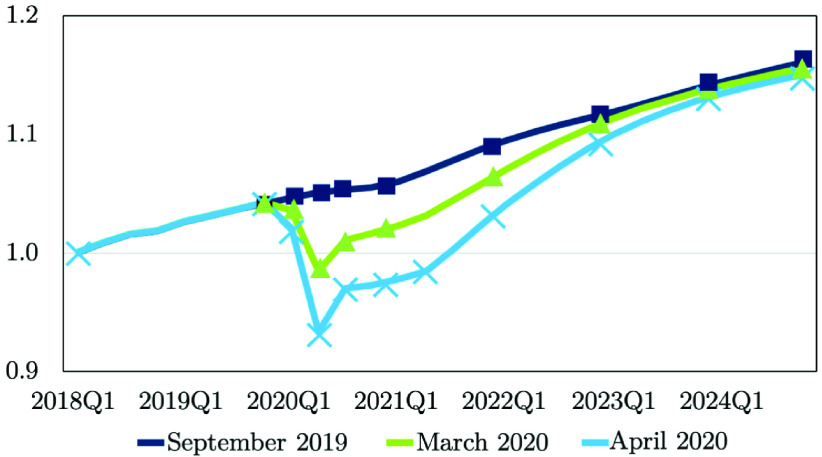
Impact of COVID-19 on the U.S. Real GDP [49].

Moody’s analytics is a trusted, leading provider of economic and financial data and forecasts for the global economy [49]. Their economic forecast reports were released on 9/9/2019, 3/27/2020, and 4/10/2020. Figure 11 depicts that the U.S. real Gross Domestic Product (GDP) has been revised downward since the vintage used in the 2020 load forecast [49]. The GDP in the first quarter of 2018 (2018Q1) was 1.0. March 2020 was the first month to significantly reflect COVID-19 impacts, and April was revised down even further [49]. Economic rebound and recovery will be dependent on the progression of COVID-19 cases as well as the medical advancements such as a vaccine. According to the Moody’s Analytics forecast [49], the potential full recovery will happen by mid 2023.

**FIGURE 12.**
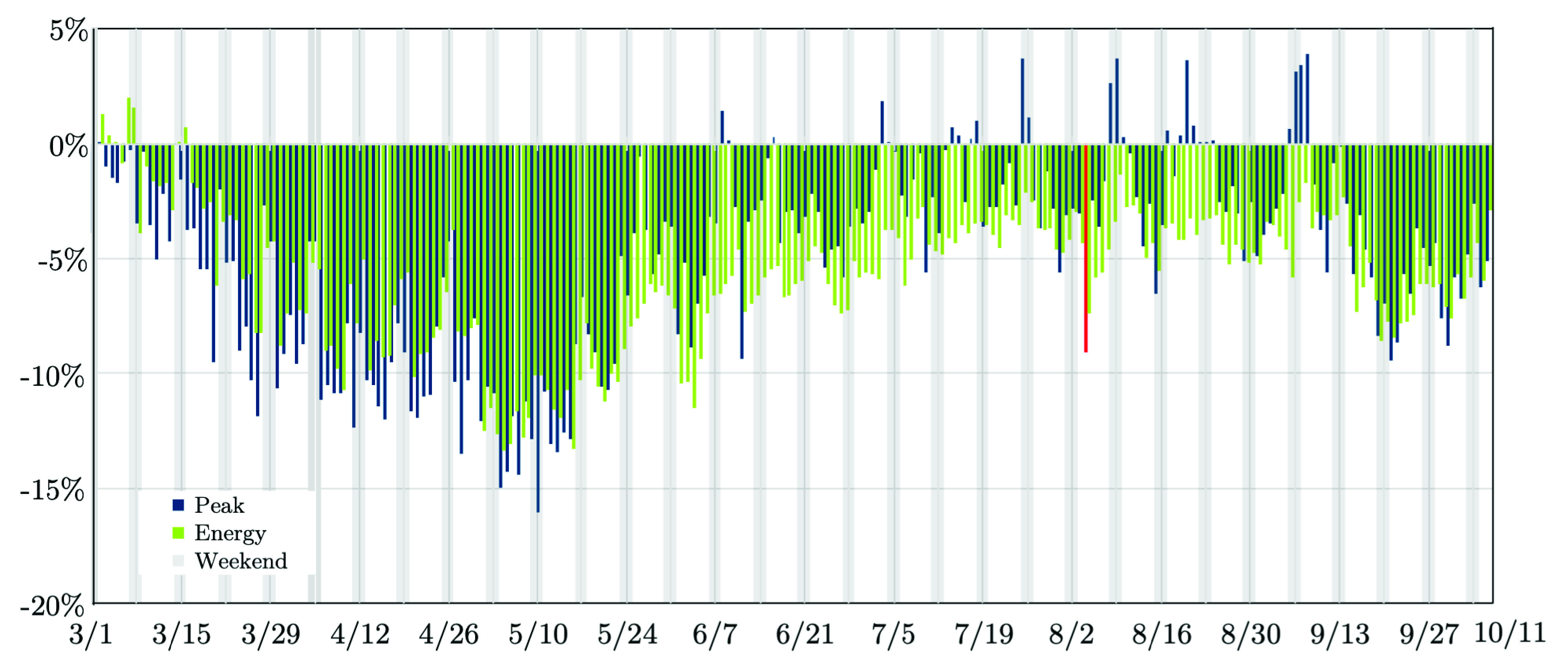
Recent-stage impact of COVID-19 on the electricity demand: The case of PJM [46].

The impacts of COVID-19 pandemic on load consumption and used energy patterns in the PJM footprint has regularly diminished since May 2020, as several phases of reopening were enforced [46]. The daily peak load is escalating back to that of a typical normal year. Electricity energy usage is also reviving, but more gradually. Figure 12 represents that the load was entirely down about 3.9% compared to 8.9% in April and May, which noted the significant influence of pandemic-related policies and social distancing practices [46]. The electrical energy usage drops in the August’s typical daily pattern, ranged from about 1.6% to 5.3%, not including Aug. 4–6 for most of the sectors, when much of the footprint endured electricity outages related to Hurricane Isaias (see the red-colored spark in Figure 12). Comparatively, the daily electrical energy usage was down throughout May 2020 anywhere from 6.1% to 13.4% [46].

**FIGURE 13.**
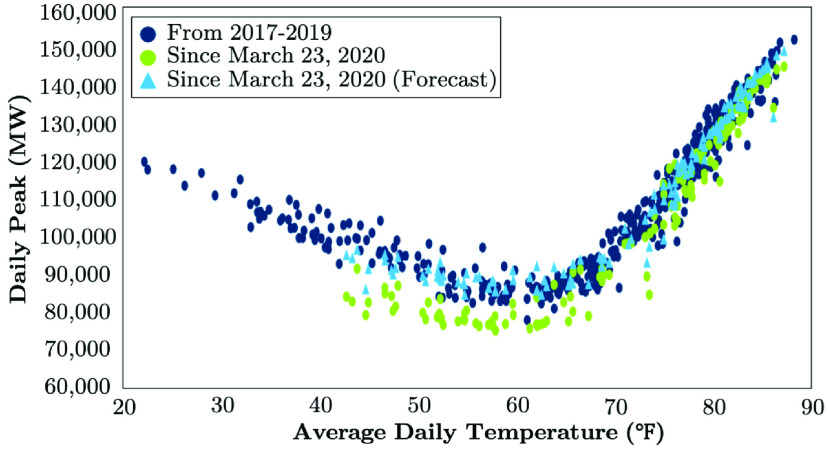
Weekday peak loads vs. ambient temperature from March 1st to September 30th, 2020: The case of PJM [46].

While the peak impacts had risen during the Summer 2020, they started to present reemerging signs as the impact of risen weather sensitivity fades. Figure 13 exhibits the positive correlation of the daily peak demand and daily temperature from March 1st, 2020 to September 30th, 2020. July to August weekday impacts were realized 1.9% less (~ 2, 600 MW) than that predicted. Through September 2020, Figure 13 indicates that the daily peak load increases to 3.4% (~ 3, 400 MW) though with noticeably larger impacts in the second half of the month. The energy impacts live larger than the peak load, but reveals the same trend. July to August energy peaks were 4.1%, ending to 4.9% through September.

#### 3) Early- and Recent-Stage Impacts of COVID-19 on Demand for Electricity: the Case of CAISO

**FIGURE 14.**
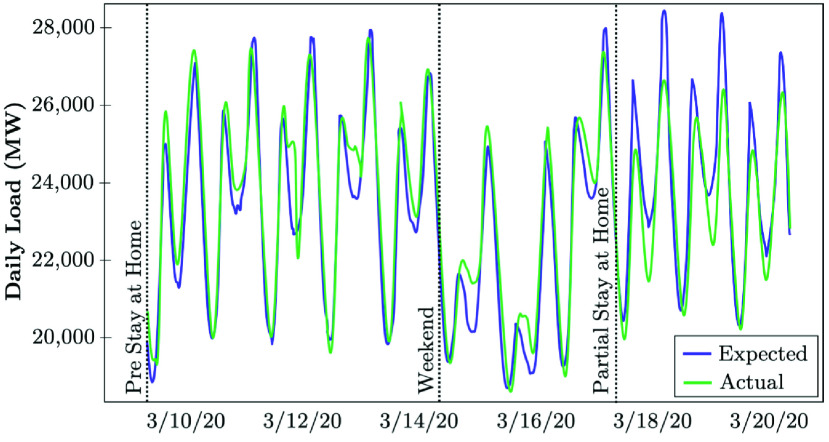
Early-stage impact of COVID-19 lock-downs on the electricity demand: The case of CAISO [50].

**FIGURE 15.**
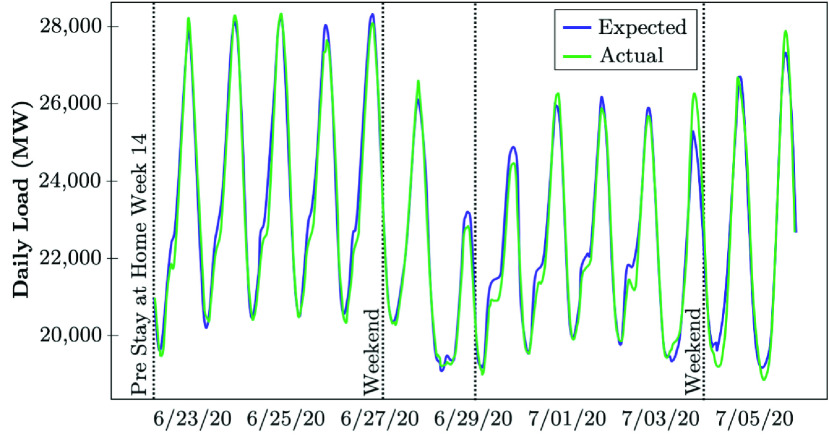
Recent-stage impact of COVID-19 lock-downs on the electricity demand: The case of CAISO [50].

Various California counties began requesting non-essential businesses to close or limit activity, including restaurants and some commercial stores, and directed companies to have their employees work from home during the early-stage evolution of COVID-19 between March 17-19, 2020. The state of California issued a stay-at-home order to all individuals living in California except for critical infrastructure sectors and essential businesses on March 20, 2020. Since the first full week of the state-wide stay-at-home order, the California ISO (CAISO) has observed the following changes in electrical demand on weekdays and weekends. CAISO utilizes the backcast analysis given the underlying weather conditions and the type of the day (see Figure 9). The load reductions are analyzed by comparing the actual realized load with the expected values if no COVID-related order were in place [50]. Figure 14 shows that the average weekday electricity consumption in California has reduced by 2.4% to 5.0% during the peak hours. The hourly average expected load differences for July range from −3.5% to +2.0%, with the most considerable reductions observed from 7 PM through 12 AM. The average load also reduced from 1.1% to 1.9% during peak hours over the weekends [50]. The state of California launched its pandemic road-map on May 9, which allowed counties to submit plans for the reopening phase of businesses, schools, and public places. While the complicated load forecast models could not have predicted the impact of stay-at-home order on the electrical load profile, the CAISO proceeded to fine-tune its models to improve the forecast accuracy in day-ahead and real-time markets as COVID-19 conditions emerged. The CAISO continued to observe that during days with warmer temperatures, less load reductions were observed in the evening ramp and peak hours (Actual), compared to the scenario if COVID-19 did not exist (Expected). Figure 15 shows that during the last week of June and the first week in July 2020, CAISO recognized a very partial load reduction (Actual) compared to the what was expected to be realized if COVID-19 was not persisting [50].

**FIGURE 16.**
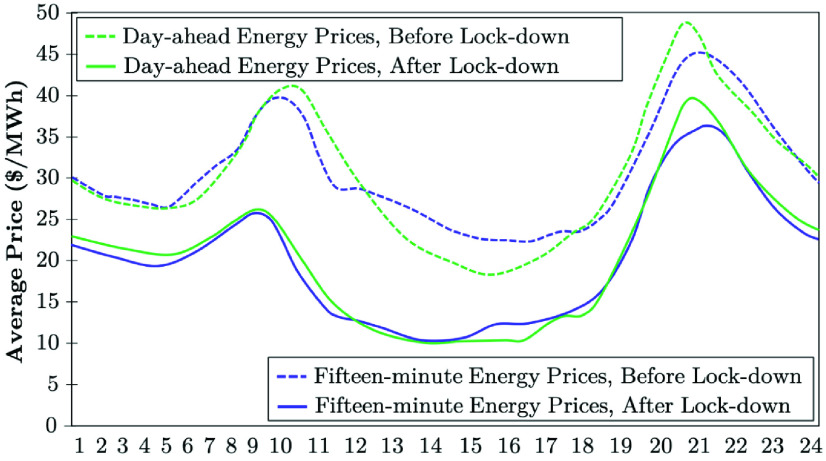
Day-ahead and fifteen-minute energy prices after and before COVID-19 lock-down: The case of CAISO [50].

Figure 16 indicates that the CAISO’s energy prices declined by about $10/MWh in the day-ahead and real-time markets after the stay-at-home orders are enforced. With higher demand for electricity since the end of May, electricity prices have increased to pre-provisioned levels [50]. CAISO has no seen major impacts on transmission and distribution grid reliability from the stay-at-home orders.

#### 4) Early- and Recent-Stage Impacts of COVID-19 on Demand for Electricity: the Case of ERCOT

**FIGURE 17.**
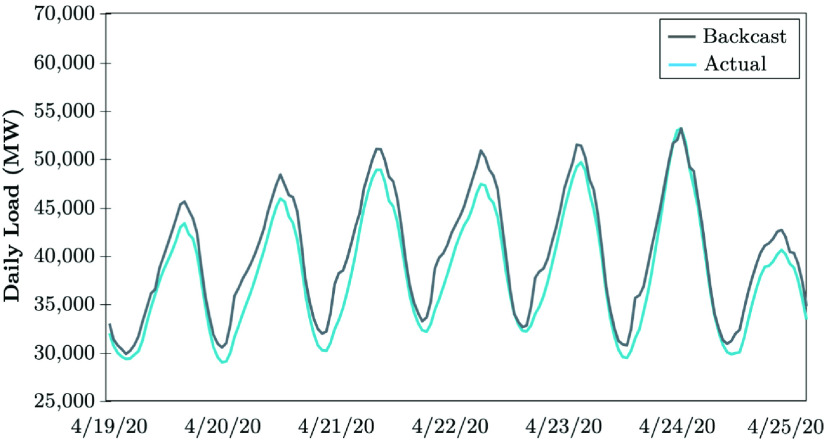
Early-stage impact of COVID-19 on the electricity demand: The case of ERCOT [51].

**FIGURE 18.**
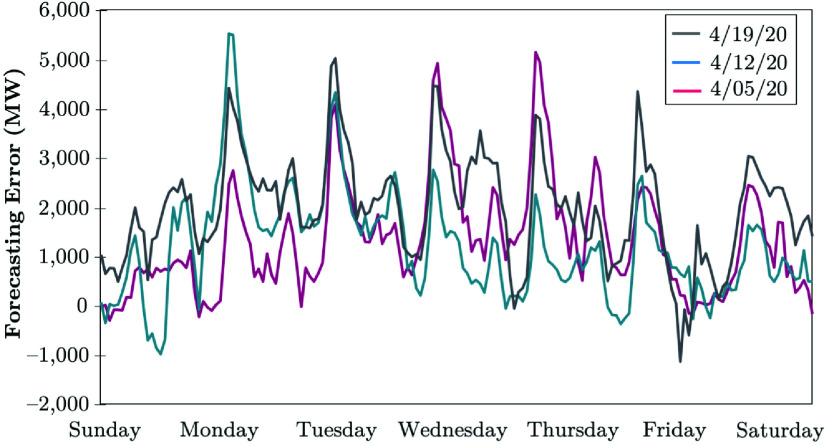
Forecasting error of COVID-19 early-stage impacts on the electricity demand: The case of ERCOT [51].

The Electric Reliability Council of Texas (ERCOT) uses a backcasting model to evaluate the load consumption, which compares the model outcomes using actual weather parameters (expected values), versus the actual hourly load realized. The difference between the actual and anticipated loads is analyzed with the error metrics. The COVID-19 impacts in April 2020 continued to lower the daily peaks, while electrical energy usage appeared to remain the same as that in the prior week [51]. Figure 17 shows that the daily peaks decreased by 4 to 5%, except on 4/24/20, the Texas hottest day in 2020. The weekly energy usage decreased by 4 to 5%, while the electrical load consumption remained consistently lower during the early morning hours between 6 AM and 10 AM. These loads are 6 to 10% lower than the normal load that the model typically predicts if COVID-19 was not in place. The largest errors observed between the backcast outcome and the actual realized electricity consumption have been occurring at 7 AM and 8 AM, shown in Figure 18. The forecasting error is calculated in (3),

\begin{equation*} \varepsilon _{Dt} = P^{b}_{Dt} - P^{a}_{Dt}. \tag{3}\end{equation*}

**FIGURE 19.**
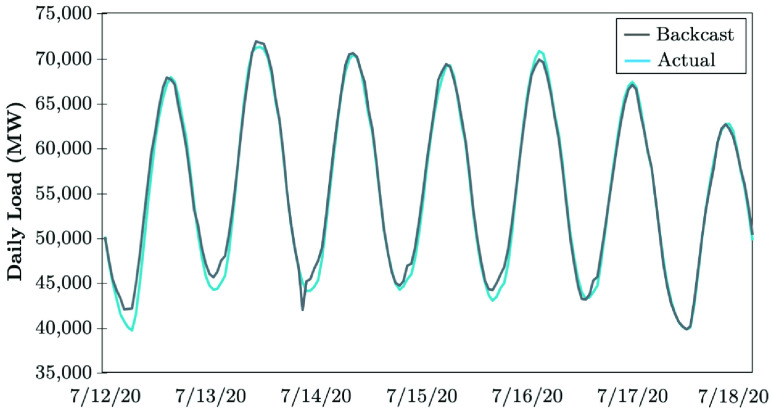
Recent-stage impact of COVID-19 on the electricity demand: The case of ERCOT [51].

**FIGURE 20.**
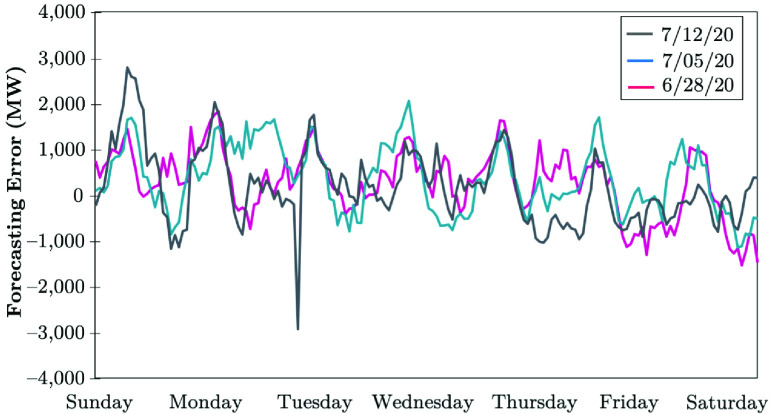
Forecasting error of COVID-19 recent-stage impacts on the electricity demand: The case of ERCOT [51].

ERCOT observes no pandemic-related impacts on the daily peak demand in the recent stage of COVID-19 evolution, while the weekly energy usage decreased by less than 1% (see Figure 19). Load reduction during the early morning hours between 6 AM and 10 AM appears to be shrinking. According to Figure 20, the largest error observed between the backcast and the actual load consumption was similar to the previous week in late June 2020. Most massive errors have been occurring at 7 AM and 8 AM.

## V. COVID-19 and Interdependent Networks

Comparing the pandemic response in 1918 to that in 2020 will not give an accurate representation of planning. Since 1918, networks have become incredibly interdependent, and crucial infrastructure is commingled together for efficiency [52]. Each of these sectors have their own challenges, but losing one sector could be detrimental for the other. For instance, many electric substations communicate with each other over the communication network. Even if the power grid stays online, losing the communication channels could be detrimental, resulting in potential loss of electricity to end-use consumers and mission-critical systems and services. In 1918, power grids were not as integrated as they are today. Many cities and towns were lighted through power islands, and they did not have interconnections. That is, an electricity outage in one area would have minimum meaningful impacts on the neighbouring cities. The way how the electric grid is structured has changed dramatically over the past decades. Losing a city or an electric substation would wreak havoc on the other surrounding grids. Even if the power demand was able to be met adequately, the system frequency could become unstable and the system would self destruct. Since 1918, the energy sector jobs have become more complex and specialized. This is the same across all industries, and it does not bode well for replacing critical employees in a crisis. This means that the top down response effective in natural disasters and crises is harder. The governors and Presidents who are responsible for managing emergency response may find themselves without the necessary skills to manage electric utilities and other critical infrastructure [53].

**FIGURE 21.**
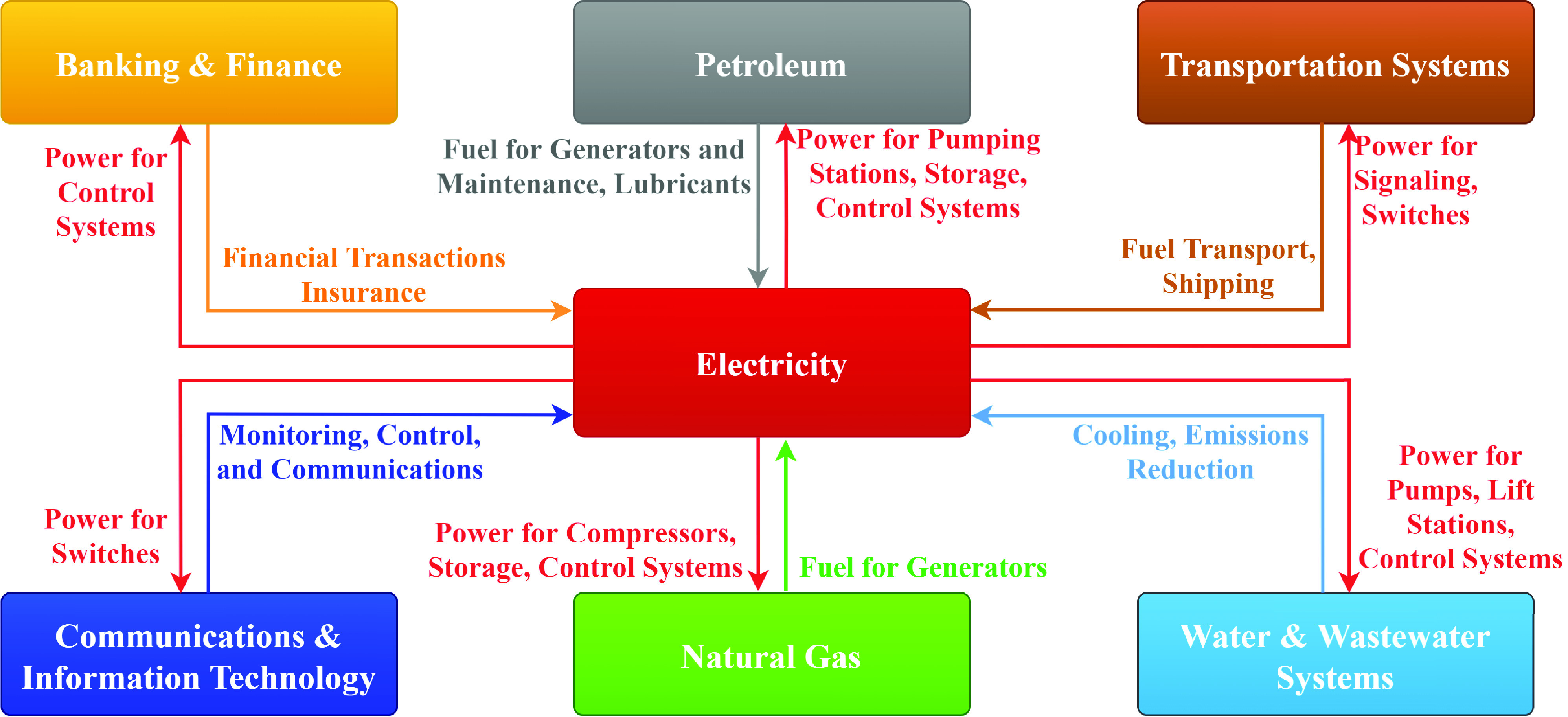
Electricity grid backbones a myriad of interdependent lifeline networks in modern societies.

The COVID-19 epidemic affected almost every single element of the human life. With the pivotal role of the electricity grid in our modern society back-boning a number of interdependent lifeline networks, exploring the impacts on some interdependent lifeline networks can shed some light on the possible consequences in power systems (see Figure 21). In this section, the disruptions caused by the pandemic on the telecommunications, water systems, supply chains, and healthcare sectors are explored with their repercussions on electric power systems.

### A. Telecommunication Network

The telecommunication (Telecom) network is a backbone connecting our society. The social distancing and stay-at-home policies resulting from the COVID-19 pandemic have highlighted the importance of this network not only for the industry but also for individuals [54]. The COVID-19 impact on telecom ranges from conspiracy theories relating 5G to the spread of COVID-19 to the necessary new connectivity infrastructure in residential homes. While absurd at the first sight, the conspiracy theory linking 5G to COVID-19 in social media platforms led to the burning of 5G towers in the United Kingdom [55].

Abruptly, the COVID-19 has sent tens of millions of white-collar workers to work remotely. Domestic and global business demand migrated from commercial to residential spaces, highly increasing the demand for mobile communications and networking infrastructure in many neighborhoods not prepared for that [56]. People working at home are part of industries, such as education and entertainment, built upon connectivity. Students and professors are at home with a clear need for high-quality internet connection to enable all types of synchronous and asynchronous online learning methods [57], [58]. With adults and kids at home, online games and streaming are another source of demand for connection [59]. However, simultaneous with this revolution, the Broadband Commission [60] has called attention to the fact that more than 3.6 billion people around the globe remain offline.

Healthcare and other industrial supply chains also heavily rely on the telecom networks to fight and adapt to the COVID-19 as it evolves [54]. Drones, cellular positioning systems, global positioning systems (GPS), radio frequency identification (RFID), and Wi-fi can be used to monitor temperature, enforce social distancing, trace infected patients, and maintain social distance [61]. The Internet of Things (IoT) is another technology which can usefully connect devices to the Internet with implications to e-commerce and its supply distribution [62]. These disruptions on telecom networks have a significant impact on power system networks. The main one is the obvious change in the electricity demand from commercial to residential sectors. The demand adjustment can be not only on the amount, but also on the time of the day that the electricity is consumed and was thoroughly discussed earlier in Section IV.

### B. Water Systems

Water system is one most energy intensive networks, an interruption in which may quickly result in a national security concern. Containment measures due to the COVID-19 pandemic have restricted people’s movements, preventing them from attending schools and workplaces and traveling, and have significantly affected their normal social life and lifestyles [63]. While in some disasters and health emergencies, water can be a vehicle for bacteriological infections, this is not the case with the COVID-19 pandemic in which freshwater is a major ally to help limit the spread of the virus. This explains the exacerbated importance to have access to potable water at home — a fundamental resource to ensure adequate health conditions and to curb the spread of the pandemic – and has modified the water consumption, outflow regime, and price.

The impact of COVID-19 on water infrastructure is especially critical in low and middle-income countries, which, even before the COVID-19 crisis, faced major water access challenges. In 2017, as discussed by World Health Organization [64], close to 30% of the world population had no safely-managed drinking water services. Furthermore, 40% of people on earth presented no access to basic hand-washing facilities with soap and water available. Last but not least, 673 million people lived in areas in conditions of open defecation. Obviously, this tragedy is more concentrated in developing nations [65].

The “simple” hand-washing action, a main measure to combat COVID-19, can be just a dream for a very significant part of the world population. Analyzing the consequences of COVID-19 in poor countries, Cooper in [66] argues that, to fix water, sanitation and hygiene (WASH) dilemmas, the design and implementation of new infrastructure policies are mandatory. Water systems are complex schemes highly dependent on electric power systems. In particular, water pumps receive a remarkable portion of the total electricity needed to run the water system infrastructure: around 4% of the total electricity usage in the U.S. is exhausted by electric pumps in water networks [67]. Investments in water infrastructure will demand support of the energy systems.

### C. Supply Chain

Supply chain (SC) response is central to battle epidemic outbreaks [68]–[70]. Cholera [71], Ebola [72], and smallpox [73] are epidemics that received sustained attention from the SC community. On March 11, 2020, the World Health Organization declared COVID-19 a pandemic [74]. Global intertwined supply networks encompass food supply, healthcare, and most service and industrial segments [59] and constitute complex production systems [75] that have been severely disrupted by the COVID-19 pandemic. Stockpiling and hoarding patterns affected the food industry during the early stages of the pandemic and resulted into a marked increase in the demand for products, such as pasta, rice, hand soap, and toilet paper [76]. Concomitantly, lock-downs and social distance created a labor shortage and logistical interruptions with repercussion on food supplies [77]. This led to a deficit of product supply in almost every segment of manufacturing industries across the world [78], which was further exacerbated by the fact that China – the world’s factory – was the early epicenter of the pandemic. The reduced circulation of cars, buses, trains, and other public transportation means is another consequence of the pandemic [79], [80]. Ride-hailing services, such as Uber and Lyft, suffered an abrupt disruption in the early stages of the pandemic [81]. Moreover, the interruption of airline flights have frozen medium and long-term distance transportation [82], [83]. Consequently, the reduction of people’s mobility severely hurt supply chains.

Disruptions in global supply chains have clear and significant consequences on the demand for electricity and power system services, ranging from power balance, voltage regulation, to network maintenance [84]. NERC announced an emergency plan addressing the supply chain risks given the manufacturing slowdown in China and the preparedness to guarantee employees safety [85]. The supply problems stemming from China have negatively influenced, not only the power systems’ equipment manufacturers, but also the renewable energy industry [86], [87]. We discuss next the impact of COVID-triggered SC disruptions on the healthcare and pharmaceutical industries.

### D. Healthcare

The COVID-19 crisis has revealed just how close to capacity most US and other countries hospitals operate, with vulnerable medical supply chains (SC) and minimal tolerance for “surge” demands [88], [89]. The health care workforce and emergency personnel are under unprecedented stress. Many front-line workers battling dual threats at work and in their home communities, while also facing economic hardship and employment insecurity [90]–[92]. The lessons of COVID-19 in systems preparedness, public outreach, and support of vulnerable communities, come at a great cost — but with the hope that they may be retained and applied to other systemic challenges in the years to come [93], [94].

The medical equipment company SC has been highly impacted by COVID-19 due to the surge in the demand for surgical masks, N95 respirators and mechanical ventilators [95]. This issue was further compounded by the prior-COVID extremely high concentration of the production of medical equipment in China, which had not enough production capacity and was struggling with the pandemic [96]. Before the pandemic, China produced 50% of the world’s face masks [97]. The increasing demand and supply issues forced the U.S. to increase and accelerate the domestic production of healthcare equipment [98], to the point that the U.S. Federal Government even ordered General Motors to produce ventilators under the Defense Production Act [99].

The pharmaceutical SC is also exposed to serious concentration risks [100]. In August 2019, only 28% of the active pharmaceutical ingredients (APIs) consumed in the US were domestically produced; China and India supplied 31% of APIs for the U.S. market [101]. The significant health security risk led to increased pressure to augment the US production of APIs and other medications [102]. Clearly, this production internalization trend in medical equipment and supplies will affect the power system infrastructure in similar ways as already discussed in Subsections V-A, V-B and V-C.

## VI. Visions for a Pandemic-Prepared Future

With the sudden shifts in electric power generation portfolios and demand profiles during COVID-19 pandemic, sustaining quality electricity supply to end customers is urgently needed. The planning, operation and control of electric power grids are impacted by “high absenteeism” pandemics in different sectors and on multiple scales. From health emergencies that could rapidly and severely limit the available workforce in electric industry for an extended and mostly unforeseeable time periods, to supply-chain disruptions leading to postponed installations and delayed maintenance implementations, the electric industry has to take all necessary actions to keep the lights on throughput the future pandemic emergencies.

The main risk factors for pandemics are *spark risk* and *spread risk*. Spark risk is concerned with how likely a pandemic is to occur in a particular area, and spread risk relates to how likely it is to spread among the populace [103]. For preparedness, it is necessary to reduce both of these risk factors. From a power grid perspective, electric utility companies were well prepared for this pandemic. By and large, there have not been any disasters, critical infrastructure failures, or other catastrophes. Thanks to previous pandemics popping up every few years, the utility companies have been well trained, and their plans were up to date. Of course, more could always be done such as drills for working from home for a week. This allows electric utilities to see what happens with their workers and verify if they have the essential tools to complete their work. In the future as more companies telework on some days of the week, this will be achieved. Cost wise, electric utilities should prioritize pandemic preparedness in the future. This means investing in keeping up to date the pandemic preparedness plans, partnering with health officials to keep prepared, and encouraging their employees to stay home if they feel sick. One main challenge with teleworking is the high dependency of the work on secure and reliable communication networks. Cybercriminals may take advantage of the crisis when more employees work remotely. Cybersecurity firms have reported a dramatic increase in spear-phishing attacks [104].

Different from the business-as-usual practices, sudden shifts in the use of electricity by the industrial, commercial, and residential sectors during the pandemics may bring about additional complexities in maintaining a continuous and reliable supply of electricity to in-need customers. Recent statistics reveal that the industrial and commercial loads have declined due to the business closures during the COVID-19 outbreak, while the residential load patterns have been trending higher as the shelter-in-place orders are in place: a slower than normal usage of electricity in the morning and increased energy use in the afternoon. Such different-than-usual changes in demand patterns may result in power distribution transformers loading capacities being exceeded. Several challenges exist in the current practices:

• Power distribution transformers are generally aged, being in service for more than 20 years, with de-rated capacities, resulting in operation closer to the limits with less energy efficiency. Existing transformers are not ready to be continuously over-stressed by the ramping in the residential electricity demand due to the evolving pandemic-shaped lifestyles.
• Transformer-customer configuration in power distribution networks was designed and planned years ago; the rising population over years and addition of new households as well as frequent feeder maneuvers and switching in power distribution network configuration have made the transformer-customer connectivity complicated or in many cases unknown.
• The existing practice does not allow recognition of “which particular household” (single- and three-phase) is being connected to “which particular transformer” (single- and three-phase connections) in the network; rather a residential “area” by a group of multiple transformers. This restricts the opportunity to control the demand as needed and limits the operational flexibility for effective transformer load management practices amid pandemics.
• The existing practices fail to holistically integrate and coordinate the objectives of feeder-transformer load management with residential household occupants’ behaviors and expected thermal quality expectations. The latter encompasses the conditions inside a building — electrical appliances, lighting, thermal conditions, ergonomics — and the impacts on the household residents’ comfort.

A robust, replicable, and scalable mechanism for real-time transformer-feeder load management during pandemics is, hence, essential; a tool that can (i) estimate the real-time residential electricity demand based on the household occupancy predictions, (ii) identify which transformer (among a network of available transformers in a distribution feeder) feeds each individual household customer, and (iii) automatically perform the transformer load management practices as needed.

**FIGURE 22.**
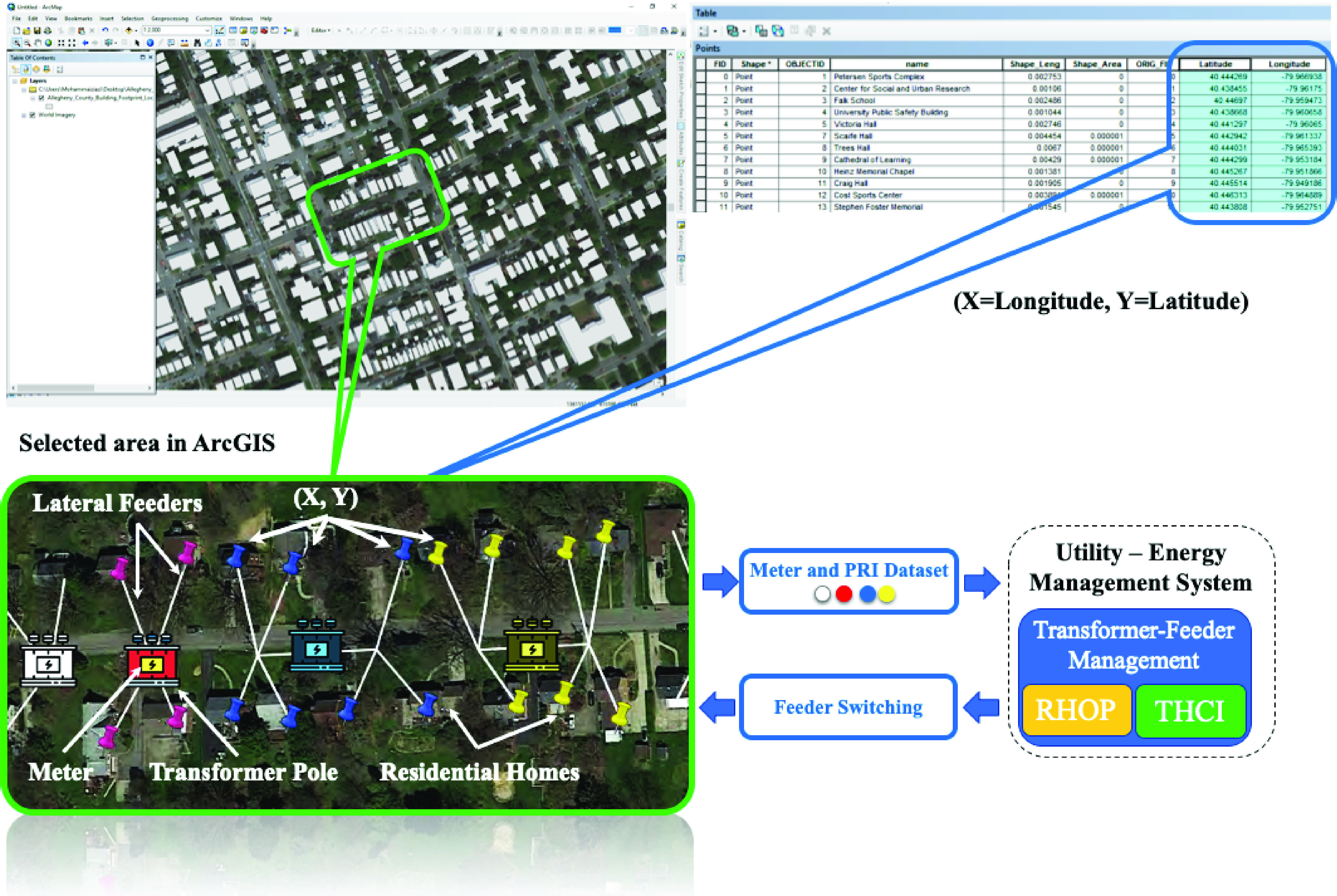
The ROADMAP platform: clustering outcome for the transformer-smart meter connectivity amid pandemics.

With the sudden shifts in the use of electricity experienced during the COVID-19 pandemic and the associated challenges it embarked on various grid operation and control practices, we suggest designing the “Real-Time Operational Awareness for Electricity Demand Management Amid Pandemics (ROADMAP)”. ROADMAP is an analytical tool that can be achieved by (i) creating a Household Occupancy and Electricity Consumption (HOEC) Dataset through field-collected data during the outbreak, (ii) developing analytics for electricity demand estimation via residential household occupancy prediction and (iii) envisioning tools for real-time transformer-feeder load management during pandemic emergencies (see Figure 22). First, a machine learning mechanism – e.g., a hybrid of convolutional neural network (CNN) and long short term memory (LSTM) – can be proposed for residential household occupancy prediction (RHOP) over a geographical area that utilizes the HOEC dataset and will allow an accurate estimation of the residential electricity demand. Second, a deep learning artificial intelligence tool can be applied for transformer-household connectivity identification (THCI); this is achieved through a comprehensive analyses of the Variational Auto Encoder and K-means Clustering methods using both the measured node voltages at the location of households and building footprint GIS information to characterize the sub-network cluster of households to their parent transformers [105], [106]. Third, when the first two modules are coupled, the real-time RHOP and THCI can initiate proactive decision making for online reconfiguration of the network so as to ensure an effective transformer-feeder load management, continuous supply-demand balance, availability of grid-support volt-var control functions, and required resource allocation.

## VII. Conclusion

In conclusion, pandemic planning is incredibly important to smooth and reliable electric power delivery. As shown through previous pandemics and the COVID-19 response around the world, the necessary ingredients for a successful response are incredibly varied. There are some key takeaways which are listed as follows:

• Absenteeism is the main issue challenging the power grid operation and control. This should be addressed in future research, especially looking at how many employees an electric utility can lose before critical functions are not able to be completed.
• Pandemic planning and preparedness documents such as those used in Canada and the U.S. are very important to successful operation of the electric utilities amid the pandemic crises.
• With many of the personnel in the electric industry not available at the workplace and with the stay-at-home orders mandating teleworking and remote connections through communication networks, a pandemic may provide an ideal environment for cyber criminals to attack the power grid. Special attention and additional investments should be made on the cyber-secured platforms in the electric industry.
• Government involvement in the pandemic response can be a very helpful factor in a successful recovery. This allows for a constant and correlated response across many interdependent industries. This is part of the reason why the European response has been observed much more effective than that in the U.S.: centralized command works in crises.

Admittedly, the full consequences of the pandemic have yet to be demonstrated. Maintenance issues may take years to surface, and budgetary challenges that have been seen universally may only pose issues in a few years. The response thus far has been measurably good, but that could change at any moment. The ability for electric utilities to be flexible is going to be crucial in the coming months and years, as the grid is going to be flexed to near its breaking point. Responsible utility management will be the only mechanism keeping the lights on. Future research should also focus on the power grid planning and operation for resilience in response to weather and cyber threats while the pandemic is in place. Multi-hazard approaches and analytics could shed lights on how to tackle the simultaneous exposure of the power grid to multiple stressors and emergencies that if not properly managed, could impeded the societal growth and result in public safety and national security concerns.
